# Review of Recent Publications in Medical Zoology

**Published:** 1892-09

**Authors:** C. W. Stiles

**Affiliations:** Washington, D. C.


### III.—REVIEW OF RECENT PUBLICATIONS IN MEDICAL ZOOLOGY.

By C. W. Stiles.

### PROTOZOA.

*Railliet et Lucet.* Note sur quelques espèces de Coccidies encore peu étudiées. Bull. d. l. Soc. d. Biol. de France, 1891, pp. 246–250.

1. *Coccidium perforans*, Lkt., 1879. Leuckart established this species for certain parasites found in the intestines of dogs, cats, rabbits and men, but several authors have expressed their doubts as to its being distinct from *C. oviforme* found in the liver. R. and L. have infected a rabbit with coccidia taken from the intestines of another rabbit, and they found upon killing the experiment-animal that the parasites inhabited only the intestines. They argue that if this species (*C. perforans*) were identical with *C. oviforme*, the parasites would have been found in the liver also, since they had every opportunity to infest that organ, but as they were not found there under these circumstances, the authors believe *C. perforans* is a true species. Other arguments for the distinction of the two species are also given. R. and L. do not, however, believe that the coccidia found in dogs and cats belong to

*C. perforans;* the parasite of the dog being *C. bigeminum* Stiles, and those of the cat, *noccidium* (?) *Rivoltæ* Grassi, (*C. Rivoltæ* belonging, perhaps, to another genus) Zürn described a parasite in the intestines of calves and pigs, which Rivolta has named temporarily *Cytospermium* (*Coccidium*) *Zürnii*, but R. and L. think it is not impossible that this species is *C. perforans.*

2. *C. tenellum.* R. et L., sp. nov., 1891. This is a parasite which the authors found in an epizootic of typhilitis among chickens; it measures but 0.021–0.025 mm. long by 0.017–0.019 mm. broad. Rivolta, Silvestrini and Mégnin have ascribed the building of diphtheritic false membranes, as well as tubercular nodules in various organs of chickens to a coccidium, but R. and L. find that this species (*C. tenellum*) is confined to the intestines. The parasites were bred and given to healthy chickens, in which they caused the disease referred to. In the Rec. d. Méd. Vét., 1891, pp. 616–621 (Typhilite coccidienne chez des poulets), the disease is discussed from a clinical standpoint. It was most fatal among chicks three to four weeks old, less dangerous to older chickens, although a number of fowls two to three months old also succumbed. The general symptoms noticed were: Dullness, loss of appetite, difficult walk and plaintive cries; the chicks generally showed constipation, occasionally preceded by diarrhœa (whitish), while the older animals were generally affected by excessive diarrhœa, sometimes bloody. Death followed a few days after the first appearance of the symptoms. The pathological lesions differed somewhat in the various animals; in all cases, however, they were confined to the coeca. Thus in the chicks which died constipated, the coeca were colored normally, but dotted with irregular whitish spots consisting of a mass of coccidia. The coeca were distended by a solid yellowish dry exudate which was filled with the parasites. Among those animals which died of diarrhœa, the cœca showed an extreme irritation—inflamed mucous, here and there ulcers. The patients were successfully treated with hyposulphite of soda, recommended by Rivolta for intestinal coccidiosis, and equal parts of fennel, aniseed, coriander, gentian, ginger, aloes, recommended by Mégnin for avian diarrhœa. These medicines were made into a cake with milk and bread crumbs.

3. *C. truncatum,* sp. nov. 1891, was found in the kidneys of the domestic goose. It measures 0.02–0.022 long by 0.013–0.016 broad. The parasite develops in the epithelial cells of the uriniferous tubes; falling into the lumen after it becomes encysted, it is expelled with the urinary products.

4. *C. bigeminum.* Stiles, 1891. When I wrote my preliminary note on this species, I was under the impression that Rivolta was the first to mention its occurrance, but my friend Railliet now corrects me, stating that its discovery is due to Fink. This parasite is described and figured in the September number of this Journal.

### TREMATODES.

Dr. P. Willach (Distomumbrut in den Lungen des pferedes; Arch. f. w. u. pr. Thierheilkunde, 1892, pp. 118–123) found in a tumor in the lungs of a horse, leaf-shaped bodies which he supposes to be rediæ (a larval stage) of a fluke, together with other structures which he interprets as fluke-eggs. It is very probable that the latter are really the eggs of some fluke, but as no figures are given of the former and the description is meagre, it is impossible to imagine what the "rediæ" (?) are. Whatever they may be, we can discard the explanation Willach gives of their probable development with scarcely a second thought. Willach thinks that either eggs or embryos of flukes were swallowed by the horse, that these had then developed into sporocysts and these latter had developed the "rediæ" which he found. Such a proceeding would be impossible, judging from our present knowledge of helminthology, for we can hardly conceive how a fluke could find the necessary conditions in a horse's lung, to run through the stages of "sporocyst" and "rediæ," larval forms which are found only in mollusca. The only stages of flukes found in horses are the adult stage (we must, of course, admit the possibility of finding "cercariæ" in early stages of infections) and eggs.

R. Leuckart read a paper before the German Zoologists on *Taenia Madagascariensis* Dav. The paper was a description of the only perfect specimen of this parasite of man which has yet come into the hands of a medical zoologist. The rostellum measures 0.1 mm. and bears ninety very peculiar hooks, resembling those of *T. tetragona* of birds. The suckers are round and rather large in proportion to the head. Proglottids numbered 500–600. The ripe proglottids begin 3 cm. back of the head and measure 2 by 1.4 mm. The uterine walls degenerate and the eggs lie free in the parenchyma; they are then surrounded by cells of the parenchyma, forming an egg-ball. The vas deferens is surrounded by prostate glands in its entire length. This character and the peculiar arrangement of the eggs in egg-balls are not known among other cestodes (Braun).

NEMATODES.

*Tricocephalus dipressiusculus*, the whip-worm of the dog has heretofore been considered as a harmless inhabitant only of the cœcum, but Mégnin (Compt. rend. d. l. Soc. d. Biol., 1892, p. 874), notes two cases of hunting dogs which died in a very anæmic condition, which was due to countless numbers of this parasite in the cœcum and in the large intestines.

Sometime ago there was considerable said in some of the European papers about an epidemic of trichinosis which occurred on board the bark "Nixe," and was at that time attributed to American pork, but a recent publication by Wasserfuhr (Deutsche Med. Wochenschrift, 1891, No. 35), contains a statement from the captain of the bark, to the effect that the American hog was entirely innocent of the charge made against him, for the epidemic was due to fresh pork purchased at the port of Iquiqui and eaten *uncooked* (!) by the sailors. Wasserfuhr insists that the trichinae in pork are killed by the Chicago process and regrets that his countrymen (Germans) insist upon eating raw pork, a custom (or "Unsitte" as he expresses it) which has long since been given up by all civilized people, except the Germans This reminds us of the speech once made by one of our prominent American physicians, who remarked in discussing the subject of trichinosis, that any one who was foolish enough to eat raw pork deserved to have trichinosis!

Prof. Janson, of Tokio, has recently published a paper, entitled "*Filaria immitis und andere bei Hunden in Japan vorkommenden Parasiten*" (Arch. f w. u. pr. Thierheilkunde, 1892, pp. 63–79), in which we find a number of observations, interesting alike to vetrinarians and zoologists. The biting louse (Trichodectes) is more common on Japanese dogs than the sucking louse (Hæmatopinus), while in Europe, the opposite is true. The acarus scab is very common, while the Scarcoptes scab is rare, also the opposite of what is found in Europe. Ticks are so common, that according to Janson, the dogs not infrequently die from the effects of their attacks. In Japan, round-worms are more frequent than tape-worms, while in Europe the latter are in the majority. It is interesting to note that a fluke (*Distoma pulmonale*), which is said to occur in the lungs of 25 per cent. of the inhabitants of certain districts, is also found in the lungs of dogs. *Tænia cucumerina*, the larval stage of which occurs in the lice and fleas of dogs, was found in 60 per cent. of the dogs examined; 50 per cent. contained *Bothriocephalus*, a tapeworm contracted from eating fish, while *T. marginata*, *T. serrata*, and *T. echinococcus* were rare. Of round-worms, *Dochmius trigon-*

*ocephalus* was present in 75 per cent., *Filaria immitis* and *Ascaris marginata* in 50 per cent., *Spiroptera sanguinolenta* in 10 per cent., while *Eustrongylus gigas* was found but twice.

The greater part of the paper is given up to a discussion of a parasite which the noted Philadelphia parasitologist described as *Filaria immitis.* The adult is found in the right half of the heart, while embryos occur, sometimes in immense numbers, in the blood. Janson states that the embryos may be found in all of the ecto-parasitic blood-sucking animals which infest the dog, and that they are also passed with the urine and other excretions, but believes we are still ignorant as to the life-history of the animal. (Note: Bancroft believes that the Trichodectes is the secondary host, C. W. S.) It is extremely interesting to note that Janson found one case where the worm-embryos passed through the placenta and infected a fœtus. Generally *F. immitis*, according to Janson, appears to do no special harm to the host, in fact, 75 per cent. of the infested dogs show no signs of infection; of the remaining 25 per cent., half are troubled only at times by the presence of the worms, while in the remainder death may occur or they, too, may recover more or less from the effects of the parasites. According to J., the infection occurs in the hot months, July–September; what then becomes of the worm is uncertain, but in January–March it reaches the heart, becomes mature, and in April–June begins to make trouble for its host. The symptoms produced by the worm vary greatly, according to the position the parasite occupies, so that a positive diagnosis can be made only by examining the blood for the embryos with a low-power (30) microscope.

In treatment Janson uses *good nourishing food*, with digitalis, squills, caffein, alcohol, æther, quinine or iron, according to symptoms.

WASHINGTON, D. C.

